# Effect of Vitamin D Supplementation on Fatigue in Multiple Sclerosis: A Systematic Review and Meta-Analysis

**DOI:** 10.3390/nu15132861

**Published:** 2023-06-24

**Authors:** Purificación López-Muñoz, Ana Isabel Torres-Costoso, Rubén Fernández-Rodríguez, María José Guzmán-Pavón, Sergio Núñez de Arenas-Arroyo, Julián Ángel Basco-López, Sara Reina-Gutiérrez

**Affiliations:** 1 Faculty of Physiotherapy and Nursing, University of Castilla La Mancha, 45071 Toledo, Spain; purificacion.lopez@uclm.es (P.L.-M.); mariajose.guzman@uclm.es (M.J.G.-P.); julianangel.basco@uclm.es (J.Á.B.-L.); 2 Research Group in Pediatric and Neurologic Physiotherapy, ImproveLab, University of Castilla La Mancha, 45071 Toledo, Spain; 3 Health and Social Research Center, University of Castilla-La Mancha, 16071 Cuenca, Spain; ruben.fernandez@uclm.es (R.F.-R.); sergio.nunezdearenas@uclm.es (S.N.d.A.-A.); sara.reina@uclm.es (S.R.-G.)

**Keywords:** supplements, disability, calciferol, cholecalciferol, neurological disorders, tiredness

## Abstract

Vitamin D supplementation has been considered a possible treatment to reduce the risk of disease activity and progression in people with multiple sclerosis (MS). However, its effect on disease symptoms remains unclear. The aim of this meta-analysis was to conduct a systematic review to assess the effect of vitamin D on fatigue in this population. The systematic review was conducted using the MEDLINE, Cochrane Library, Embase and Web of Science databases from inception to May 2023. Randomized controlled trials (RCTs) reporting pre–post changes in fatigue after vitamin D supplementation were included. Pooled effect sizes and 95% confidence intervals (95% CIs) were calculated by applying a random effects model with Stata/SE (Version 16.0; StataCorp., College Station, TX, USA). The Preferred Reporting Items for Systematic Reviews and Meta-Analyses guidelines were followed. A total of five studies with 345 individuals (271 females; age range: 25.4–41.1 years) were included. A significant reduction in fatigue was perceived when vitamin D supplementation was compared with a control group: −0.18 (95% CI: −0.36 to −0.01; I^2^ = 0%). Thus, our findings show that the therapeutic use of vitamin D on fatigue in people with MS could be considered. Nevertheless, due to the lack of agreement on the dose to be applied, it is recommended to use it under medical prescription.

## 1. Introduction

Multiple sclerosis (MS) is an autoimmune and inflammatory chronic disease of the central nervous system that constitutes one of the leading causes of disability among young adults [1]. Multiple sclerosis can produce a variety of symptoms, such as fatigue, blurred vision, optic neuritis, weakness, dizziness, balance disturbances, cognitive decline, and problems with bladder control, as well as an increased risk of depression and anxiety [2]. Fatigue is one of the most common and disabling symptoms [3] and can be described as a subjective lack of physical and/or mental energy that interferes with usual activities [4]. In people with MS, fatigue can be central and peripheral, and both types can occur simultaneously. Central fatigue is related to dysfunctions of the central nervous system, especially processes of inflammation, demyelination, and/or neurodegeneration, and peripheral fatigue is related to non-specific factors of the disease or dysfunctions of other body systems [5,6]. Otherwise, fatigue can cause decreased physical activity and concentration, memory disturbances, executive difficulties and feelings of tension, anxiety, or sadness [7]. Furthermore, it is frequently perceived by people as the most debilitating symptom that significantly affects quality of life [7,8]. There are a number of drug treatments for MS-related fatigue; however, to date, there is insufficient evidence to support which ones are most effective [7,9]. 

Although the etiology of MS is still uncertain, it is likely that the interaction between genetic and environmental factors, along with others, contributes to its appearance [10,11]. Some factors, such as the duration and intensity of sunlight exposure and high-latitude geographical areas, are correlated with the incidence and prevalence of MS [11]. This connection could be due to low ultraviolet radiation exposure and low vitamin D (VD) status in these areas [12,13,14]. 

Vitamin D is a fat-soluble steroid hormone produced predominantly in response to ultraviolet B (UV-B) irradiation of the skin [15]. The main forms of VD in the diet are ergocalciferol (vitamin D2) of vegetable origin and cholecalciferol (vitamin D3) of animal origin. Vitamin D appears to have an immunomodulatory effect that includes the activation and proliferation of lymphocytes, the differentiation of T cells, and a reduction in inflammatory cytokines [10]. Some studies have confirmed the association between low serum levels of 25-hydroxyvitamin D (25(OH)D) and the risk of MS onset, also constituting a risk factor for disease activity and progression in early stages [10,16]. Likewise, it has been observed that suboptimal levels of VD can contribute to inflammation and axonal degeneration in people with MS [17]. These associations and their effects on immune and central nervous system cells raise the question of whether vitamin supplementation could be used as a therapeutic strategy in MS [11]. Therefore, VD supplementation is an area of great interest because it is a potentially modifiable environmental factor for the development of MS and a possible treatment to reduce the risk of disease activity and progression [15]. However, to date, consensus clinical guidelines on the use of VD in MS do not offer clear recommendations on its effect on the progression and activity of the disease [18,19,20]. The most studied clinical variables in this regard with controversial results are relapse rate [10,14,21,22,23], disability or disease progression [10,14,17,23,24], and the appearance of new magnetic resonance imaging (MRI) lesions [23,25,26]. While some studies have not found a significant positive effect of VD in relation to the relapse rate and disease progression [10,14,17,21,22,23,25], others, such as the study of Camu et al., [26] did find one. On the other hand, regarding the appearance of new MRI lesions, VD has been shown to have a significant positive effect in several studies [23,24,26], although in the Cochrane review by Jagannath et al. [17], this effect was not found. In contrast, the effect of VD supplementation on fatigue has been poorly studied and remains uncertain [27], and considering that fatigue is one of the most disabling symptoms and the one with the greatest impact on the quality of life of people with MS, it seems pertinent to investigate possible treatments that improve this variable. 

Thus, the aim of this systematic review and meta-analysis was to synthesize the evidence from clinical trials and to estimate the effect of VD administration on fatigue in people with MS.

## 2. Methods

The Cochrane Collaboration Handbook [28] and the Preferred Reporting Items for Systematic Reviews and Meta-Analyses (Table S1) [29] guided the present study. The protocol was registered in the PROSPERO database (CRD42023400524). 

### 2.1. Data Sources and Searches

Two reviewers (R.F.-R. and SR-G) independently searched the MEDLINE (via PubMed), Cochrane Library, Embase (via Scopus), and Web of Science (WoS) databases from inception to May 2023. The databases were reviewed to identify randomized controlled trials (RCTs) aimed at determining the effectiveness of VD supplementation on fatigue in people with MS. No language restrictions were applied. Moreover, the reference list of the selected studies and the list of references of other systematic reviews and meta-analyses were reviewed for additional relevant studies. The Mendeley desktop find and merge duplicates tool was employed to search for duplicates, and a third reviewer peer-reviewed the search process (M.J.G.-P.). Further details of the search strategy used for each database are available in Table S2.

### 2.2. Study Selection

The search criteria according to the PICOS strategy were as follows: (i) Participants: people with MS; (ii) Intervention: VD supplementation; (iii) Comparison: no intervention, participants treated with a placebo, or with another intervention that also received an intervention group; (iv) Outcome: fatigue; and (v) Study design: RCTs. 

The exclusion criteria were as follows: (1) studies where VD intervention could not be isolated, and (2) studies not reporting enough data to calculate effect size.

### 2.3. Data Extraction

Two authors (P.L.-M. and A.T.-C.) independently extracted the following information from each included study: (1) first author name and publication year; (2) country; (3) sample characteristics: sample size (female), mean for age, body mass index (BMI), disease severity, type of MS and disease duration, and baseline level of VD; (4) intervention characteristics: duration, frequency and dose of the intervention, adherence, and side effects; and (5) outcomes: fatigue scale. The authors attempted to contact corresponding authors to request information on missing data from the studies and, when this was not possible, the study was excluded. Disagreements in data extraction were resolved by consensus.

### 2.4. Classification of the Disease, Baseline Level of 25(OH) D, and Outcome

For the characteristics of the disease, we extracted the severity and duration of MS. The disease severity was reported through the Expanded Disability Status Scale (EDSS), considering score between 0 and 5 as mild EDSS and a score ≥ 5 as severe EDSS [30,31], and the total baseline value of the scale was reported. For the duration of the disease, the time since diagnosis was selected because it was the most common in the included articles. 

Studies reported the 25(OH)D baseline level in ng/mL or nmol/L, and for analyses, the nmol/L unit was converted to ng/mL. 

Fatigue was measured through one or more self-report questionnaires. When the fatigue scale was subdivided by domains, we used the total score for the analyses. When studies applied more than one test for reporting an outcome, a combined estimate was calculated. Moreover, when studies were inversely scaled (i.e., lower values indicating worse outcomes), the mean in each group was multiplied by −1.

### 2.5. Risk of Bias Assessment 

Two researchers (S.R.-G. and M.J.G.-P.) independently assessed the risk of bias of the included studies using the Cochrane Collaboration’s tool for assessing risk of bias (RoB2) of RCTs [32]. Any disagreements were resolved by consensus or by discussion with a third reviewer (A.T.-C.). The RoB2 tool assesses risk of bias according to five domains: (i) randomization process, (ii) deviations from intended interventions, (iii) missing outcome data, (iv) measurement of the outcome, and (v) selection of the reported result. Overall bias was rated as (i) “low risk of bias” if the study was classified as “low risk” in all domains, (ii) “some concerns” if at least one domain was scored as “some concerns”, and (iii) “high risk” if there was at least one domain rated as “high risk” or several domains as “some concerns” that could affect the validity of the results. 

### 2.6. Data Synthesis

Random effect models were used to estimate the pooled standardized mean differences (SMDs) and their 95% confidence intervals (95% CIs) of the effectiveness of VD supplementation on fatigue in people with MS. 

According to the Cochrane Handbook recommendations, we extracted the pre–post mean, standard deviation (SD), and sample size of each arm of the trials. For those studies that did not report these data, we collected the mean difference and standard error (SE) or SD of the change. In addition, when data were given as % relative change, this was applied to baseline measurements, and the effect size was calculated considering similar SD.

Statistical heterogeneity between studies was examined using the I^2^ statistic. I^2^ values of 0–40% were assumed to indicate “not important” heterogeneity, 30% to 60% represented “moderate” heterogeneity, 50% to 90% represented “substantial” heterogeneity, and 75% to 100% represented “considerable” heterogeneity. We accordingly considered their corresponding *p*-values and 95% CIs [33]. To assess the robustness of summary estimates and to detect whether any individual study accounted for a large proportion of the heterogeneity, sensitivity analyses were performed, and influence graphs were generated by removing the included studies one by one from the analyses. Likewise, meta-regression models—considering age, % of females, BMI, baseline EDSS, % of people with relapsing-remitting type, and baseline VD level—were conducted to determine their influence on the estimated effect. Finally, publication bias was assessed via the visual inspection of funnel plots and Egger’s regression asymmetry test to assess the effects of small studies [34]. All statistical analyses were performed using StataSE v. 15 (StataCorp, College Station, TX, USA).

## 3. Results

### 3.1. Literature Search

**Figure 1:**
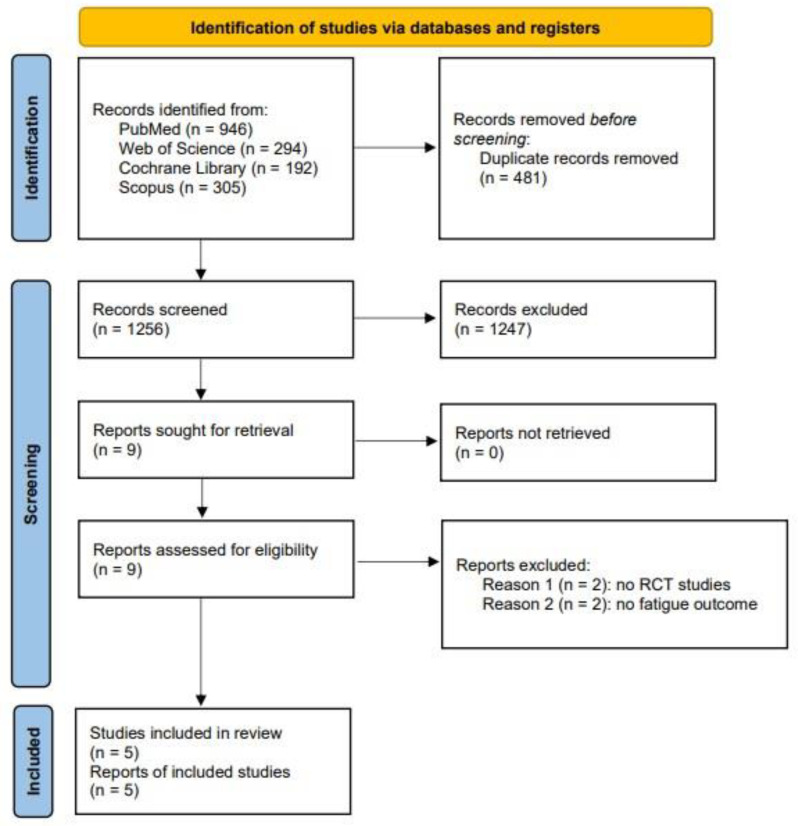
PRISMA flow diagram.

A total of 1256 studies were identified through the systematic searches, of which 481 duplicated records were removed (Figure 1). Finally, after a full-text review of the nine studies assessed for eligibility, five studies were included in the systematic review, and five provided data for the meta-analysis [35,36,37,38,39].

### 3.2. Study Characteristics 

The main characteristics of the included studies are available in Table 1. All of them were RCTs. The country of origin of the studies was heterogeneous: one was conducted in Iran [36], one in Israel [35], one in Norway [37], and two in the Netherlands [38,39]. The total number of participants included among the studies ranged from 38 to 158. Concerning the characteristics of the participants, a total of 345 participants were considered for the final analysis, of which 271 were females. The age range for the included participants was between 25.4 and 41.1 years, and one study showed the BMI status of the participants. Participants were categorized as overweight (25–29.9 kg/m^2^) [37] according to their BMI. Disease severity was mild in all studies, and the mean duration ranged from 5.7 months to 11 years. Finally, most participants were relapsing–remitting patients.

### 3.3. Interventions

Vitamin D supplementation varied across the included studies from 1 mcg to 50.000 IU. Additionally, the frequency (times per week) ranged from 1 to 7 in half of the studies [36,37], and the dose was daily, while in the other half, the dose was once a week [35,38,39]. Finally, the intervention length ranged between 8 and 96 weeks.

Of the included studies, one [36] had four arms (two interventions (VD and VD + aerobic training) and two controls (placebo and aerobic training). For this study, we compared VD with placebo and VD + aerobic training with aerobic training for the analyses. In the study of Kampman et al. [37], the intervention group included VD + 500 mg calcium/day, and the control group included placebo + 500 mg of calcium/day.

The studies assessed the effect of VD supplementation through different fatigue scales. The most common fatigue scale used was the Fatigue Severity Scale (FSS) [37,38,39]. 

### 3.4. Meta-Analysis

**Figure 2:**
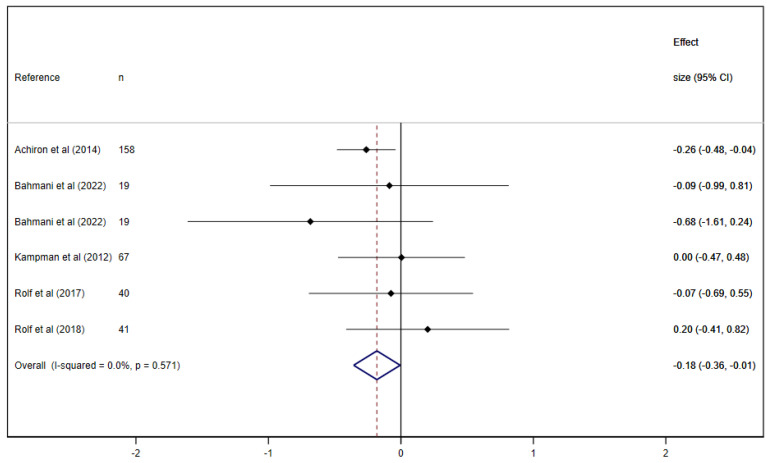
Pooled estimated effect size of the association between vitamin D supplementation on fatigue in people with multiple sclerosis (RCTs [35,36,37,38,39]).

When exploring the effect of VD supplementation on fatigue in people with MS (RCTs), there was a significant reduction in fatigue −0.18 (95% CI: −0.36 to −0.01; I^2^ = 0%) (Figure 2).

### 3.5. Sensitivity Analyses, Meta-Regression Models, and Publication Bias

The sensitivity analyses indicated that, in general, there was no change in the direction or significance of the overall effect of VD supplementation on the analyzed outcome when any of the included studies were omitted. The global effect estimator of VD also remained significant on fatigue when any of the included studies were removed, except for that published by Achiron et al. [35]. When we removed this study, the effect became not significant −0.05 (95% CI: −0.33, 0.24). Meta-regression models revealed no significant role of age, % females, BMI, baseline EDSS level, % of people with relapsing–remitting type, and baseline VD level on the fatigue outcome analyzed (Table S3). Finally, no publication bias was detected (*p* = 0.551) (Figure S1).

### 3.6. Risk of Bias Assessment 

The overall risk of bias assessment for RCTs showed that two studies (40%) presented high risk [36,39], and three studies (60%) were classified as low risk [35,37,38]. Further details according to the score of each item for the risk of bias are available in Figure S2.

## 4. Discussion

Vitamin D has been administered as a supplement for decades in people with MS since its deficiency can be a pathogenic risk factor and influence the activity of the disease [40,41]. However, to our knowledge, this is the first systematic review with meta-analysis that synthesized the effects of VD supplementation in relation to fatigue, a symptom that affects most people with this disease. Our data show a significant reduction in fatigue in those who received VD supplementation compared to the control group. 

In recent years, growing interest has emerged in the potential beneficial effect of VD supplementation on fatigue in people with MS, but the results are controversial. A wide 2018 Cochrane review [17] that evaluated the benefit of VD supplementation to reduce disease activity in relation to fatigue only included the studies of Achiron et al. [35] and Kampman et al. [37], showing contradictory and inconclusive results. The same results were obtained in a 2020 umbrella review [42] that analyzed the evidence for dietary interventions in MS and, regarding fatigue, only included the two mentioned studies [35,37]. On the other hand, in the cross-sectional study of Albrechtsen et al. [43], the intake of VD along with omega-3 fatty acids also showed a trend toward a reduction in fatigue. Similar to this last study, our study data from five clinical trials showed a positive effect of VD supplementation on fatigue in this population. 

The study of Achiron et al. [35] showed the best results in terms of a significant reduction in fatigue in the VD group in relation to the control compared to the other studies, which generally showed positive trends or no effect on fatigue. The characteristics that differentiate the study of Achiron et al. from the others are a larger sample size with its 158 participants representing nearly 50% of the total sample and a dose of VD of 280 IU/week (1 mcg/day), much lower than that used in the rest of the studies, which ranged between 20,000 IU [37] and 98,000 IU [39] per week.

Currently, there is no consensus on the optimal dosing of VD intake as adjuvant therapy in MS [10]. People with MS seem to have reduced serological and metabolic responses to VD supplements, which suggests that they may need higher doses than others to achieve clinically relevant effects [44,45]. Supplementation with high doses of VD is generally well tolerated by people with MS [15,46,47]. However, some studies recommend supplementing with VD only in cases of confirmed deficiency [16,48] as well as not exceeding 600 IU/day, since higher doses could increase the risk of toxic side effects [12,16,49,50]. In the meta-analysis by McLaughlin et al. [15], high doses were associated with worse outcomes in general and were even reported to potentially increase the relapse risk. In contrast, other studies suggest that higher doses are more effective than lower doses [16], as reported by a recent cross-sectional study that found a positive association with improvement in quality of life and fatigue [46]. Without having conclusive data on what the optimal dose is for people with MS, it would be advisable to follow the recommendation of consulting with healthcare providers to obtain personalized guidance on VD supplementation according to the specific circumstances and the medical history of each individual [19].

It is necessary to emphasize that the baseline level of 25(OH)D is a very important parameter to consider since VD supplementation is more effective when applied to subjects with low basal levels [51,52]. Although in our meta-regression analysis there were no significant differences in this regard, it is worth highlighting that in most of the studies that provided data, the participants had a baseline normal 25(OH)D level [53], which may have been the reason why no differences were found. Moreover, this may also have influenced the fact that the effect of VD supplementation was not greater.

Furthermore, there is currently interest in disorders of sphingolipid (SL) metabolism in MS, as they play an important role in the regulation of the immune response and inflammation [54,55]. Recently, fingolimod has been used for the treatment of MS, which is an immunomodulatory drug that targets the sphingosine-1-phosphate receptor and helps reduce inflammation and prevent damage to the myelin sheath [56]. Although in the present meta-analysis, only one study reported that some participants were taking this medication [38], it is possible that variations in SL levels among study participants could influence the efficacy of VD supplementation in alleviating fatigue symptoms, as VD is known to affect SL metabolism [57].

Some limitations that might limit the robustness of our estimates should be acknowledged, such as the small sample size of the included studies. Nonetheless, in this systematic review and meta-analysis, a significant effect of VD on fatigue was detected, although RCTs with larger samples would be necessary to confirm these findings. Furthermore, there was wide variability in the duration of the treatment and the dose of VD used among studies, so further research is necessary to determine the optimal dose to improve fatigue in people with MS and understand the possible benefits and risks associated with these variables. On the other hand, due to the limited data about the type of VD administered, a complementary analysis could not be made to determine its influence on the results. In this regard, most of the studies that provided data used vitamin D3 [37,38,39], which appears to be more effective than vitamin D2 in increasing serum levels of 25(OH)D [58]. Finally, some studies suggest that factors such as sun exposure could act as confounding variables when determining the influence of VD on fatigue [41], since in some studies, sunlight exposure was more strongly associated with fatigue than 25(OH)D concentrations [27,59], a factor that was not considered in our study.

In conclusion, this systematic review and meta-analysis suggests that supplementation with VD might have a significant effect on reducing fatigue in people with MS. Nevertheless, due to the lack of agreement on the dose to be applied, it is recommended to use VD under medical prescription. Future research to understand the optimal dose and duration of the treatment and studies in samples with lower baseline levels of 25(OH)D are needed to optimize clinical outcomes.

## Figures and Tables

**Table 1:** Characteristics of the studies included in the meta-analysis.

Study Characteristics			Population Characteristics						Intervention Characteristics						Outcome
First Author (Year)	Country	n (Female)	Age (Years) Mean ± SD	BMI (kg/m^2^) Mean ± SD	Disease Severity (EDSS) Mean ± SD	MS Type	Disease Duration (Years) Mean ± SD	25(OH)D Baseline Level (ng/mL)	Duration/w	Frequency(x/Week)	Vit D Dose	Comparator	Adherence	Side Effects	Fatigue Scale
Achiron et al., (2014) [35]	Israel	158 (118)	41.1 ± 9.2	NR	2.9 ± 2.6	RR: 91.7%	6.2 ± 5.5	NR	6 months	7	1 mcg	Placebo	IG: 90%	IG: headache and dizziness	FIS; MFIS
													CG: 91%	CG: abdominal pain	
Bahmani et al., (2022) [36]	Irán	38 (38)	AT + VitD: 27.70 ± 2.68	NR	3–5 (range)	NR	NR	AT + VitD: 25.80 ± 1.81	8	1	50,000 units	AT Placebo (CG)	AT + VitD: 100%	NR	MFIS
			AT: 26.77 ± 2.27					AT: 26.55 ± 1.50					AT: 90%		
			VitD: 25.44 ± 2.29					VitD: 26.44 ± 1.42					VitD: 90%		
			CG: 28.11 ± 3.62					CG: 27.20 ± 3.45					CG: 100%		
Kampman et al., (2012) [37]	Norway	68 (48)	IG: 40 (21–50) **	IG: 28 (21–41) **	IG: 2.5 (0–4.5) ***	RR: 100%	IG: 11 (1–27) **	IG: 55.56 (46.87; 64.26) nmol/L ****	96	1	20,000 IU + 500 mg calcium/day	Placebo + 500 mg calcium/day	IG: 100%	No adverse events	FSS
			CG:41 (26–50) **	CG: 26 (18–40) **	CG: 2.0 (0–4.5) ***		CG: 10 (2–26) **	CG: 57.33 (48.37; 66.28) nmol/L ****					CG: 91.67%		
Rolf et al., (2017) [39]	Netherlands	40 (26)	IG: 38.5 ± 7.8	NR	IG: 2.0 (1.5–2.5) *	RR: 100%	IG: 7.5 (4.4–11.7) months *	IG: 58 (38–82) nmol/L *	48	7	7000 IU first 4 weeks and 14,000 IU up to week 48	Placebo	IG: 90.91%	NR	FSS
			CG: 37.6 ± 9.6		CG: 2.0 (1.5–2.3) *		CG: 5.7 (3.9–11.7) months *	CG: 53 (43–63) nmol/L *					CG: 92%		
Rolf et al., (2018) [38]	Netherlands	41 (41)	IG: 38.6 (28.0–45.0) *	NR	IG: 2.0 (1.4–2.0) *	RR: 100%	IG: 3.8 (2.8–11.4) *	IG: 85 (71–111) nmol/L *	16	7	4000 IU	Placebo	IG: 92%	IG: headache and dizziness	FSS
			CG: 35.1 (33.0–45.0) *		CG: 2.0 (1.0–2.5) *		CG: 5.4 (1.2–7.9) *	CG: 78 (68–95) nmol/L *					CG: 72.41%	CG: abdominal pain and stomach discomfort	

AT: home-based aerobic training, BMI: body mass index, CG: control group, EDSS: Expanded Disability Status Scale, FIS: Fatigue Impact Scale, IG: intervention group, IU: international units, mcg: micrograms, mg: milligrams, MFIS: Modified Fatigue Impact Scale, n: sample size, ng/mL: nanograms per milliliter, nmol/L: nanomoles per liter, NR: not reported, NA: not available, RCT: randomized clinical trial, RR: relapsing–remitting, SD: standard deviation, VitD: vitamin D, w: weeks, x: times per week, 25(OH)D: 25-hydroxy vitamin D, * = median (IQR); ** = mean (range); *** = median (range) **** = mean (95% CI).

## Data Availability

The dataset that supports the findings of this study is available from the corresponding author on reasonable request.

## Supplementary Materials

The following supporting information can be downloaded at: https://www.mdpi.com/article/10.3390/nu15132861/s1. Figure S1: Funnel plot showing publication bias results for fatigue, Figure S2: Risk of bias for randomized controlled trials of vitamin D interventions, Table S1: PRISMA 2020 checklist, Table S2: Search strategy for each database, Table S3: Meta-regression analyses.
